# An Innovative Assessment Framework for Remote Care in Orthopedics

**DOI:** 10.3390/healthcare13070736

**Published:** 2025-03-26

**Authors:** Flaviu Moldovan, Liviu Moldovan

**Affiliations:** 1 Orthopedics—Traumatology Department, Faculty of Medicine, George Emil Palade University of Medicine, Pharmacy, Science, and Technology of Targu Mures, 540142 Targu Mures, Romania; 2 Faculty of Engineering and Information Technology, George Emil Palade University of Medicine, Pharmacy, Science, and Technology of Targu Mures, 540142 Targu Mures, Romania; liviu.moldovan@umfst.ro

**Keywords:** orthopedic surgery, remote care, sustainability, hospital, environment, indicator

## Abstract

**Background/Objectives**: Orthopedics is a medical specialty that is experiencing a significant increase in the volume of interventions due to an ageing population. By activating remote care pathways, orthopedic surgeons can contribute to improving environmental sustainability. The aim of this research is to develop assessment tools for remote care pathways in orthopedic surgery, inspired by advanced practices of international hospitals. **Methods**: The research methods consisted in identifying the key areas that make up the remote care pathways in orthopedic surgery and designing appropriate indicators to assess their sustainability. Their levels of achievement were designed by collecting from the literature the most advanced practices reported by hospitals worldwide. The practical validation of the developed model was performed at an orthopedic hospital. **Results**: Based on the recommendation of the College of Surgeons, we have identified four key areas: remote consultations in orthopedics, incentives for staff travel options, involvement of surgical patients, and minor surgical interventions. In each of these areas, we have designed an indicator, for which we have described the levels of achievement on a scale from 1 to 5. The indicators are also rated for their importance on a scale of 1 to 5, according to the extent to which they influence the achievement of the sustainability objectives. The practical implementation of the developed model at an orthopedic hospital has shown its suitability for the purpose of the research and its compatibility with the certifications held by the hospital. We computed the sustainability indicator in each area as the product between the achievement level and the importance of the indicator. The sum of the four indicators provides the global sustainability indicator. The fulfillment degree of the requirements related to remote care pathways in orthopedic surgery is obtained by reporting the actual value of the global sustainability indicator to the maximum value, which in the explored situation is 61.42%. To have high efficiency for improvement measures we have used the Eisenhower matrix. It is evidenced that the highest priority must be given to the indicator incentives for staff travel options. **Conclusions**: Implementation of the system in healthcare facilities promotes continuous improvement of remote care pathways in orthopedic surgery, improves environmental sustainability, and thereby contributes to reducing climate change.

## 1. Introduction

Climate change is having a negative impact on health, constituting the greatest global threat to health in the 21st century [1]. Orthopedic surgeons can support efforts to improve environmental sustainability and, thus, contribute to efforts to reduce climate change [2]. Staff and patient travel to hospitals contribute up to 18% of total carbon emissions from health-related activities [3].

An important strategy to reduce the carbon footprint in the healthcare sector is telemedicine [4,5]. For certain categories of patients and interventions, telemedicine involves consultations by telephone or videoconferencing [6]. This includes patients who do not require a careful physical, internal, or visual examination [7]. All these categories of patients are likely to be able to perform remote consultations if they can communicate via remote technology [8,9]. Remote consultations, in certain situations where appropriate, also reduce travel for medical staff [10,11]. Low-carbon, pre-targeted transportation options such as electric cars, bicycles, or walking can also be integrated [12]. González et al. [13] proposed a new model of orthopedic-traumatology healthcare that is based on virtual consultations.

In the configuration of orthopedic healthcare services, emphasis should be placed on disease prevention. This facilitates the reduction of avoidable hospital admissions and also the performance of invasive, resource-intensive surgeries [14]. Reducing hospital lengths of stay and maximizing patients’ fitness for surgery can be achieved by promoting healthy behaviors such as smoking cessation and exercise [15]. Further measures that facilitate the provision of environmentally sustainable healthcare and that are more cost-effective and increase patient satisfaction are measures that promote the relocation of care closer to the patient’s home [16]. They consist of the establishment of extended primary care clinics where non-hospitalized patients are treated and can be managed by general practitioners or nurses. In this way, endoscopies, minor surgery, or day surgery can be performed [17].

The Royal College of Surgeons of England has published concrete guidance on the criteria for selecting suitable patients for remote consultations [18]. The recommended solutions for care pathways and travel, which require further exploration, include the development of policies for remote consultations, incentives for staff to use low-carbon travel options, education campaigns for surgical patients, and the development of outreach clinics for minor surgery.

Based on these recommended solutions, revealed by the specialized literature regarding the remote care pathways in orthopedic surgery, we formulated the following research question:

What are the current practices and appropriate indicators for evaluation of remote care pathways in orthopedic surgery?

The aim of our research is to develop an assessment tool for remote care pathways in orthopedic surgery, inspired by advanced practices of hospitals around the world.

The assessment tool consists of a system of indicators and the qualitative and numerical evaluation methods used to evaluate them.

## 2. Materials and Methods

In this study, we have used the following research methodology:

- Research design;
- Design of the key domains for remote care in orthopedics;
- Data collection and analysis from the scientific literature in the medical field and extracting the most relevant and recent aspects related to remote care in orthopedics that have been implemented by international hospitals;
- Elaboration of the contents and evaluation grids of the indicators for the remote care in orthopedics, where it is used as inputs for the information collected from the analysis of the scientific literature;
- Validation in practice of the developed theoretical model at an orthopedic emergency hospital.

### 2.1. The Research Design

The research was designed to investigate the research question posed in the Section 1. The Ethics Committee of the Targu Mures County Emergency Hospital (ECHTM) [19] approved the conduct of the study. The research stages, in terms of data collection, development of the theoretical model, and its validation in the practice of the orthopedic hospital, were carried out in accordance with the ethical principles of the Declaration of Helsinki.

### 2.2. The Key Domains for Remote Care Pathways in Orthopedic Surgery

Through a literature review and discussion among the authors, we felt that the most relevant way in establishing the key areas of the remote care pathways in orthopedic surgery was to follow the recommendations of professional associations of surgeons. In this way, we selected the 4 key areas, which are recommended by the Royal College of Surgeons of England’s good practice guide [20], and are as follows: remote consultations in orthopedics, incentives for staff travel options, involvement of surgical patients, and minor surgical interventions. To measure sustainability within orthopedic surgery remote care pathways, it is necessary to develop a specific indicator in each of the four selected areas: (I1) remote consultations in orthopedics; (I2) incentives for staff travel options, (I3) involvement of surgical patients, and (I4) minor surgical interventions. They align with the Sustainable Development Goals of the World Health Organization. The development of the evaluation tool was carried out in a multi-step process by a team of experts consisting of the head of the orthopedic department, primary care physicians, orthopedic specialists, residents, and management experts.

### 2.3. Data Collection and Analysis

**Figure 1:**
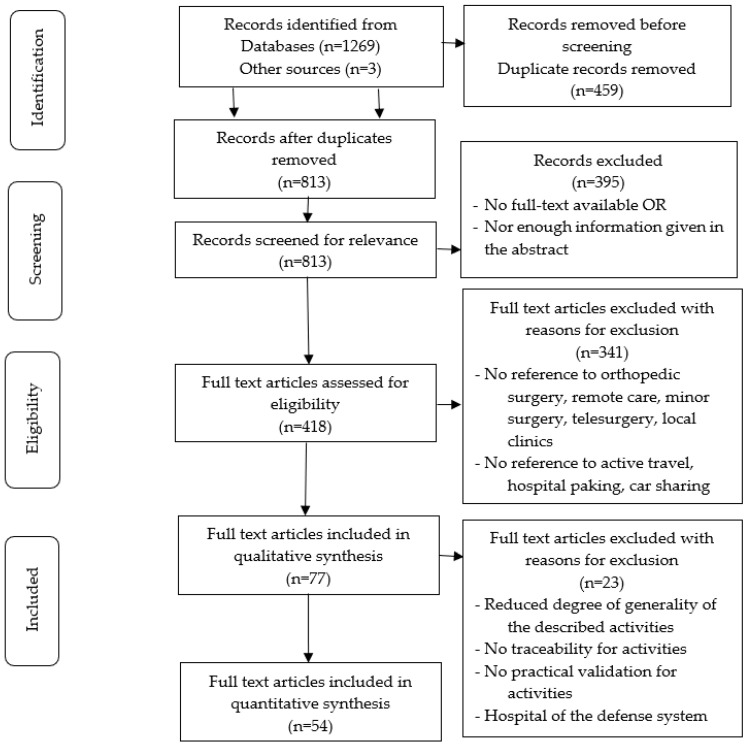
The PRISMA flow diagram details the systematic review for search and study selection process.

We continued our research by selecting and extracting from scientific literature the most advanced practices related to remote care pathways in orthopedic surgery, using the PRISMA guidelines: preferred reporting items for systematic reviews and meta-analyses (Figure 1).

We searched relevant medical databases such as Medline (via Ovid), Embase, Cochrane, PubMed, and the Scopus and Web of Science databases. For the search, we used the following keywords: remote care or orthopedics or surgery, minor surgery or local clinics or telesurgery, and active commuting or hospital parking or car sharing. Articles were selected that presented evidence-supported advanced practices, published in the last 5–10 years in English.

From the articles selected for the study, we extracted the contents of the activities that support the design of remote care pathways in orthopedic surgery, as well as the barriers that hinder their implementation. The information was systematized in the 4 key areas and was used to design the related indicators: remote consultations in orthopedics, incentives for staff travel options, involvement of surgical patients, and minor surgical interventions.

### 2.4. Validation in Practice of the Developed Theoretical Model

We continued the research with the experimental part. The objective was to validate in practice the 4 indicators at the Orthopedics-Traumatology Department at the Targu Mures County Emergency Hospital (ECHTM).

The team of auditors that conducted the practical testing of the theoretical model was composed of the orthopedic department head, the hospital quality assurance officer, the orthopedic head assistant, and an orthopedic resident.

## 3. Results

### 3.1. Indicators Contents and Evaluation Grids for the Key Areas

We continued the study by using advanced practices extracted from the 54 literature references for the descriptions of the indicator levels.

#### 3.1.1. The Key Area Remote Consultations in Orthopedics

In the research by Donaghy et al. [21], a remote care pathway solution was presented by setting up local clinics in which orthopedic surgeons assess fractures and provide care for musculoskeletal problems. The rapid contact with orthopedic consultants, elimination of travel, and time savings were positively appreciated by patients [22]. Bridging the healthcare access gap can be achieved through regulatory policies, technology integration, privacy measures, and patient education [23].

Orthopedic surgeons should offer virtual visits as an alternative to in-person assessments [24]. Evidence regarding the use of telemedicine in orthopedics indicates safety, cost-effectiveness, validity in clinical evaluations, high patient, and orthopedic physician satisfaction [25]. Another consultation method for patients requiring orthopedic spine care is telephone calls [26].

Grandizio et al. [27] show that in upper extremity and hand surgery, telemedicine applications via videoconferencing offer benefits to patients. The use of videoconferencing for orthopedic consultations in the remote clinic is also suitable for inpatients in emergency rooms, outpatient visits, and postoperative care [28]. Buvik et al. [29] show that video-assisted remote consultation can be safely offered to orthopedic patients.

Expanding telehealth services and increasing the number of telehealth sessions requires updating telehealth equipment [30] and integrating modern technologies [31]. The virtual fracture clinic is a safe, patient-centered means of providing trauma care, reducing unnecessary clinic attendance, and is cost-effective [32]. A telehealth orthopedic fracture clinic employing a virtual clinician was reported by Snoswell et al. [33].

Alongside all these reports of remote care pathways, two major forms of tele-orthopedics can be identified. These are telemonitoring via teleconsultation and telemetry and telerobotic and telementoring telesurgery [34]. Sahu et al. [35] investigated shoulder range-of-motion assessment in a telehealth environment. In the absence of additional safety data, whenever possible, orthopedic surgeons should maintain a low threshold for follow-up and physical assessment of patients [36].

With the support of these practices, the remote consultations in orthopedics indicator was elaborated (Table 1).

#### 3.1.2. The Key Area Incentives for Staff Travel Options

Developing a sustainable transport infrastructure is effective in promoting active travel [37]. Encouraging active travel behavior is possible with the support of behavioral theories [38]. Active travel is a basic indicator of activity that contributes to individual and social health and well-being [39]. The proximity of hospitals to bus routes and bus stops induces an increased likelihood of the proportion of active commuting trips [40]. Healthcare facilities should be in high-density residential areas serving a high number of patients [41].

The development of physical features is associated with a decrease in the proportion of trips by private car [42]. Facilitating bicycle travel can be supported by new traffic control devices such as bicycle boxes [43]. Awareness of environmental responsibility correlates with the choice to travel by bicycle [44].

The most popular form of transportation for medical staff and patients is travelling by private car [45]. However, a large part of the costs involved are generated by hospital parking [46]. Parking policy relaxation can be achieved through new intelligent parking management infrastructure [47]. This is associated with an increase in the number of trips made by cars [48].

A new mode of transport is car sharing [49]. But to increase user satisfaction, it requires optimization of routes and schedules [50]. The car-sharing industry is still in its early stage of development [51]. The factors that hinder the development of car-sharing systems are economic, technological, behavioral, and regulatory [52]. Of these, increasing public awareness and education is imperative [53].

With the support of these practices, the incentives for staff travel options indicator was elaborated (Table 2).

#### 3.1.3. The Key Area Involvement of Surgical Patients

In patients undergoing primary total knee replacement surgery, Timmers et al. [54] used as a standard part of care an app that provides valuable information about the preoperative phase, surgery, and recovery. In joint replacement, the use of mHealth apps facilitates recovery and opens new possibilities for patient care [55]. An electronic health app developed by Pronk et al. [56] guides patients in pain management and opioid use.

In clinical care, when there is transparency about how personal health information is used, digital health consent is facilitated [57]. It is also able to address data quality issues [58]. Implementing a digital consent system requires careful interface design [59].

Patient involvement is useful in decision-making at the departmental level [60]. Shared decision making improves service delivery and governance [61].

Non-pathologic tissues are minimally damaged by microdecompression techniques associated with small-incision procedures [62]. For minimally invasive decompression of the cervical spine, the use of cervical microendoscopic foraminotomy and cervical microendoscopic diskectomy procedures is safe and effective [63].

New surgeons have a poor perspective on their non-technical skills [64]. Surgical aptitude is also facilitated by surgeons’ non-technical skills, which has direct effects on surgical performance.

With the support of these practices, the involvement of surgical patients’ indicator was elaborated (Table 3).

#### 3.1.4. The Key Area Minor Surgical Interventions

All surgical procedures performed under local anesthesia in the outpatient setting constitute minor surgery [65]. Minimally invasive orthopedic surgery is based on small skin incisions, reduced muscle dissection, improved visibility with endoscopy and fluoroscopy, application of special instruments, and robotic assistance [66,67].

By increasing confidence in technical as well as non-technical skills, mid-level general surgery residents can independently perform outpatient procedures without jeopardizing patient safety or satisfaction [68,69]. In the management of orthopedic trauma, a widely applied concept is biological osteosynthesis [70].

Minor surgery is an effective aid in the initial diagnosis of lesions referred to for evaluation in primary care [71]. Once the results are analyzed and compared with similar studies of minor surgery performed in primary care, there is a high correlation between clinical diagnosis and histologic findings [72].

In primary care, due to high anatomopathologic concordance, patients favorably receive minor surgery [73]. Electrosurgery is a technique that patients consider safe and satisfactory, especially for head lesions [74].

The efficiency of office-based minor surgery compared with performing the procedures in other specialized centers can be demonstrated by analyzing the variables: type of procedure and surgery, clinical/pathological correlation, early surgical complications, and cost per procedure [75]. The minor surgery program in primary care decreases the cost compared with the specialty surgery program [76].

With the support of these practices, the minor surgical interventions indicator was elaborated (Table 4).

#### 3.1.5. Levels of Importance for Indicators

The designed indicators may have diverse levels of importance for healthcare facilities. To quantify this aspect, we introduced a second variable, named “Importance of the indicator”, which is also evaluated on five levels, as follows:

1. Unimportant: the indicator has low importance and does not affect the achievement of the sustainability objectives related to remote care pathways in orthopedic surgery;

2. Low importance: failure to comply with the requirements of the indicator could slightly affect the achievement of the sustainability objectives;

3. Important: failure to comply with this requirement formulated by the indicator could negatively affect the achievement of the sustainability objectives;

4. Particularly important: failure to comply with the requirement of the indicator could compromise the achievement of the sustainability objectives;

5. High importance: failure to comply with the indicator requirement may even compromise the achievement of sustainability objectives.

Placement in these levels is based on the auditors’ assessment, supported by criteria such as the content of the organizational objectives related to remote care pathways in orthopedic surgery, the method of measuring the objectives and their degree of achievement, the potential for improvement of the quality management system, the number and type of non-conformities recorded in the previous audit, and the progress recorded by the healthcare facility in implementing corrections and corrective actions, etc.

### 3.2. Indicator Matrix and Continuous Improvement Cycle

We developed the indicator matrix for remote care pathways in orthopedic surgery (Table 5), which provides an overview of the indicators designed for the evaluation of the healthcare facility.

**Figure 2:**
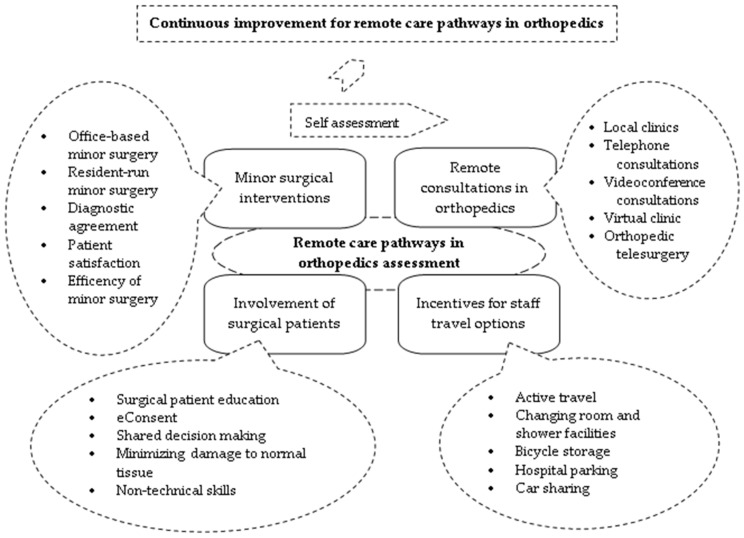
The assessment framework model for remote care pathways in orthopedic surgery.

By aggregating these results into the theoretical model of the continuous improvement cycle, we developed the assessment framework model for remote care pathways in orthopedic surgery (Figure 2).

The evaluation framework brings together the four key areas of the remote care pathways in orthopedic surgery, which are covered in the sequence of planning-doing-verifying-acting. The progress recorded in each evaluation cycle is quantified by the four indicators through the five related levels of achievement, which are represented with broken lines in the outer callouts.

### 3.3. Practical Validation of the Theoretical Model

This section presents the results of the developed model practical validation. It was conducted at the ECHTM orthopedic hospital. The assessment results are registered in the self-assessment tool for remote care pathways in orthopedic surgery (Table 6).

By comparing the indicators’ requirements (Table 1, Table 2, Table 3 and Table 4) with the situation in the ECHTM orthopedics department, the auditors assessed the achievement levels of the four indicators in Table 6 (column “Level (Li)”). Then they assessed the importance of the indicators (column “Importance (Ii)”) according to the extent to which they affect the achievement of the sustainability objectives related to remote care pathways in orthopedic surgery (as formulated in Section 3.1.5). Finally, the sustainability indicator (Si) was calculated as the product between the achievement level (Li) and the importance (Ii) of the indicator.

**Figure 3:**
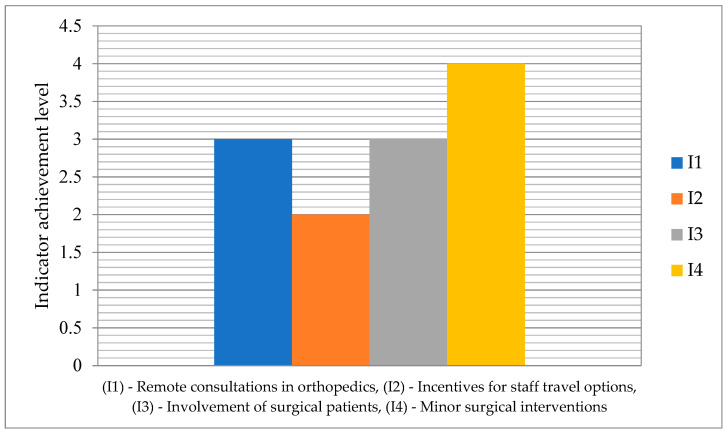
Achievement level of indicators for the remote care pathways in orthopedic surgery.

In Figure 3 the achievement level of indicators related to remote care pathways in orthopedic surgery, on the scale 1–5 is depicted.

Indicator I2 (incentives for staff travel options) has the lowest level 2, while indicator I4 (minor surgical interventions) has the highest level 4. No indicator reaches the maximum value of 5.

**Figure 4:**
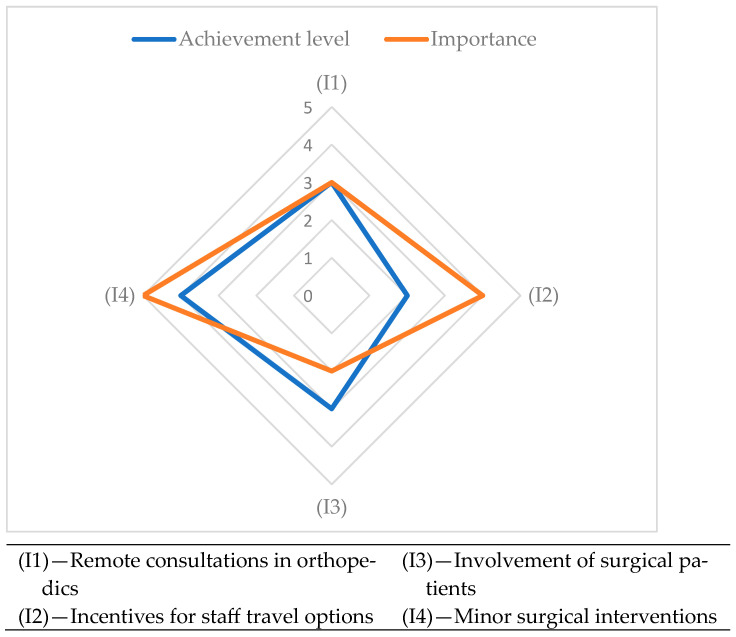
The remote care pathways in orthopedic surgery evaluation graph.

In Figure 4, the evaluation graph for correlation between the achievement level and importance of the 4 indicators assessing the remote care pathways in orthopedic surgery is depicted.

The sum of the individual remote care pathways indicators (Table 6) is the global sustainability indicator (GS_RCP_) [77]:

(1) $${\mathrm{GS}}_{\mathrm{RCP}}=\overset{4}{\underset{i=1}{\sum }}S_{i}=\overset{4}{\underset{i=1}{\sum }}L_{i}\cdot I_{i}=43$$

The maximum value for the remote care pathways in orthopedic surgery indicator (GSmax_RCP_) is obtained by summing up the maximum values 5, of the 4 individual indicators:

(2) $${\mathrm{GSmax}}_{\mathrm{RCP}}=5\cdot \overset{4}{\underset{i=1}{\sum }}I_{i}=5\cdot 14=70$$

The fulfillment degree of the requirements related to remote care pathways in orthopedic surgery (FDR_RCP_) is obtained by reporting the actual value of the global indicator to the maximum value:

(3) $${\mathrm{FDR}}_{\mathrm{RCP}}=\frac{{\mathrm{GS}}_{\mathrm{RCP}}}{{\mathrm{GSmax}}_{\mathrm{RCP}}}\cdot 100\;=\frac{\;43}{70}\cdot 100=61.42\%$$

**Figure 5:**
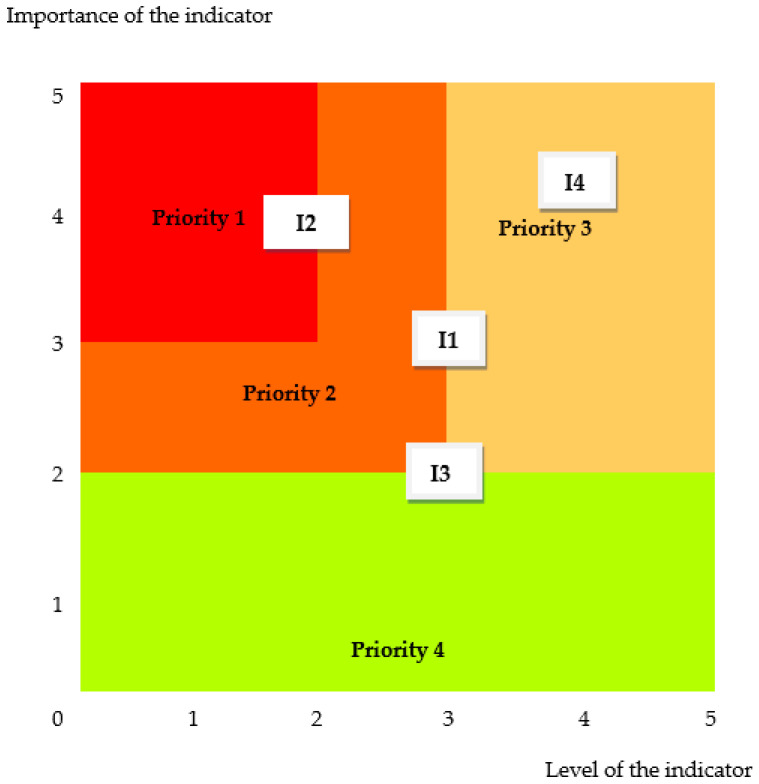
The prioritization framework for remote care pathways in orthopedic surgery: (I1) remote consultations in orthopedics, (I2) incentives for staff travel options, (I3) involvement of surgical patients, (I4) minor surgical interventions.

The fulfillment degree of the requirements related to remote care pathways in orthopedic surgery reflects the extent to which the performed activities in the orthopedics department cover the levels described by the four indicators, but by adopting improvement measures, it can be increased. To have high efficiency, we have prioritized the adopted measures using a prioritization framework (Figure 5).

It prioritizes the measures to improve the indicators as they are positioned in its quadrants, between high priority (1) and low priority (4). The highest priority must be given to the indicator (I2) incentives for staff travel options.

## 4. Discussion

In this study, we found that the indicators designed for the evaluation of remote care pathways in orthopedic surgery in the four key areas—remote consultations in orthopedics, incentives for staff travel options, involvement of surgical patients, and minor surgical interventions—are suitable for the purposes of the research. We assessed that they met the requirements for the evaluation of remote care pathways in orthopedic surgery in the four key domains indicated by the Royal College of Surgeons of England. We also found that the indicators are compatible with the frameworks implemented in hospitals, through the requirements formulated in the national legislation concerning the accreditation of outpatient healthcare units [78], as well as those with beds [79]. The system developed in this research is also compatible with the European framework for quality assessment in hospitals DUQuE [80].

A glossary of quality and health-specific terms could facilitate their practical application. This has the potential to facilitate mutual understanding between auditors and auditees. With this support, the evaluation methodology has the potential to guide healthcare facilities and health personnel towards sustainable development [81,82].

Another finding of our study shows that indicator (I2) incentives for staff travel options, need to be addressed as a priority. For this, the development of a bicycle car park including safety elements is necessary. A new intelligent parking management infrastructure would facilitate the relaxation of the parking policy.

The findings of the study conducted by Shodi et al. [83], show that remote physiological and therapeutic monitoring have increased exponentially for the management of chronic medical diseases in patients. In orthopedics, it is used for the management of total joint arthroplasty. To make the monitoring process as effective as possible, in addition to these results, we found that, along with digital tracking applications, there is a need to incorporate remote physiologic monitoring devices during the total postoperative knee arthroplasty period.

Findings from studies by Rizan et al. [84] and Wormer et al. [85] on sustainable medical interventions indicate long-term benefits. However, in our study, the economic efficiency of sustainable surgical interventions could not be demonstrated. We believe that this is due to the way expenses are recorded in the healthcare facility’s accounting, which does not allow for easy identification. Therefore, there would be a need for separate expenditure headings dedicated to sustainability and the existence of criteria for apportioning mixed expenditure. We believe that remote care pathways in orthopedics are only effective if sustainability is balanced with cost-effectiveness, along with other factors that enter medical decision-making.

Several studies, such as that of Eckelman et al. [86], report durable interventions that have been in operation for more than a decade. In our study, we found that these are not widely available in orthopedic wards. We are of the opinion that durable interventions should not be analyzed as singular elements detached from the clinical context. Theoretical study should be followed by analysis of the effectiveness of their large-scale implementation in clinical practice. In order for stakeholders, such as managers, surgeons, and patients to support their implementation, they first need to accept them. In this way, the results will be more effective and long-lasting.

Based on the data obtained, we consider that the strengths of the study consist of the development of a self-assessment tool that is composed of a system of indicators as well as their qualitative and numerical evaluation methods. This constitutes an innovative approach that complements the existing literature through the way in which the content and evaluation methods of the indicators were designed. The study also offers an innovative and concrete methodology for implementing remote care pathways in orthopedics in key areas that, until now, have only been indicated but have not been studied in detail by professional associations of orthopedic surgeons. The findings of this study can be applied in clinical practice by including the innovative system of indicators in the current frameworks implemented in hospitals, in accordance with the requirements formulated in the national legislation on accreditation [78,79], as well as those of the European framework for quality assessment in hospitals [80]. The application of the research results in clinical practice alongside existing frameworks is easy, due to their compatibility. In this way, significant insights into clinical practice are provided, as well as improved clinical practice.

The practical challenges of Implementing the system proposed in this research face a series of barriers, which we consider to be like those identified in the study conducted by Scott Kruse et al. [87] for the implementation of telemedicine solutions worldwide. These are specific to the country, the organization, the patient, and the medical staff. The practical implementation problems refer to cost barriers and reimbursement of expenses (13%), assimilation of technical problems by the staff (11%), resistance to change (8%), the patient’s level of education (5%), and the patient’s age (5%), as well as other barriers with weights below 4%.

The limitations of this study relate primarily to the results obtained in the literature exploration phase. Although we used a robust search strategy, we may not have identified all advanced practices in the medical field. Consequently, the indicators may not contain some relevant medical practices. The dynamics of the field, which is constantly evolving, may contribute to this. Another limitation concerns the assessment of the effectiveness of the interventions collected. The results are reported from cross-sectional studies, in which we collected retrospective data, which provide initial evidence. The inclusion of longitudinal studies in the research allows us to follow the evolution of some measures and to assess their effectiveness as well as their economic efficiency.

Validating the model in a public orthopedic hospital induces another limitation. Further validations of the model in orthopedic hospitals with different forms of organization, public or private, will allow a wider applicability of the indicators in the field of orthopedics or even to other medical specialties.

For future research directions, we have identified advanced database searching, collection, and systematization of the contents of successful medical practices, allowing for the expansion of indicator descriptions. Given the rapid evolution and advanced digitization of medical processes, future research should be oriented towards longitudinal studies evaluating the long-term effectiveness and efficiency of sustainable interventions in orthopedics. In turn, the evaluation method should be digitized through the development of appropriate software, which would simplify its applicability and ensure process traceability.

## 5. Conclusions

Remote care pathways in orthopedic surgery are realized in four key domains: remote consultations in orthopedics, incentives for staff travel options, involvement of surgical patients, minor surgical interventions. Each indicator has five levels defined, which makes it possible to measure the degree of achievement on a scale from 1 to 5. The indicators have different importance, which are also quantified on a scale from 1 to 5, according to how they contribute to the sustainability objectives of remote care pathways in orthopedic surgery. The pairs of values and degree of fulfillment importance of the indicators allow the representation in an Eisenhower matrix that prioritizes the improvement measures related to remote care pathways in orthopedic surgery.

The strengths of this study consist of the development of the self-assessment tool that is composed of the four indicators as well as their qualitative and numerical evaluation methods. This study provides an innovative and concrete methodology for implementing remote care pathways in orthopedics in key areas that, until now, have only been indicated but have not been studied in detail by professional associations of orthopedic surgeons. The findings of this study can be applied in clinical practice by including the innovative system of indicators in the current frameworks implemented in hospitals, in accordance with the requirements formulated in the national legislation on accreditation, as well as those of the European framework for quality assessment in hospitals. In this way, significant insights into clinical practice are provided as well as the orientation of hospitals and staff towards sustainable development.

## Figures and Tables

**Table 1:** The indicators for (I1)remote consultations in orthopedics.

Indicator Level	Name of the Indicator Level	Indicator Level Description
1	Local clinics	Setting up local clinics where orthopedic surgeons provide care for musculoskeletal problems and fracture assessments.
2	Telephone consultations	Surgeons offer consultations by phone call as part of a dedicated service. Smartphone apps are being developed to enable real-time teleconsultations.
3	Videoconference consultations	In the remote clinic, videoconferencing is used for orthopedic consultations. They are used for inpatients, emergency room patients, outpatient visits, and postoperative care.
4	Virtual clinic	Telehealth services are expanded by upgrading telehealth equipment and integrating modern technologies. A virtual orthopedic fracture clinic is implemented, and a virtual doctor is employed.
5	Orthopedic telesurgery	The two major forms of tele-orthopedics are implemented: telemonitoring through teleconsultation and telemetry and telerobotic telesurgery and telementoring.

**Table 2:** The indicators for (I2) incentives for staff travel options.

Indicator Level	Name of the Indicator Level	Indicator Level Description
1	Active travel	Behavioral theories that encourage active travel are promoted. Walking and taking the bus are encouraged, and there are bus routes and bus stops in the vicinity of the hospital.
2	Changing room and shower facilities	Staff lockers are adapted to support active travel. Shower facilities are provided.
3	Bicycle storage	There are safety features for traveling by bicycle. The hospital operates a bicycle parking lot that is accessible and includes safety features.
4	Hospital parking	Hospital management engages in traffic management in the congested and increasingly congested areas in the hospital neighborhood. A new intelligent parking management architecture facilitates the relaxation of parking policy.
5	Car sharing	By increasing public awareness and education, car sharing is promoted as a new mode of transportation. Car-sharing industry, route optimization, and scheduling are developed.

**Table 3:** The indicators for (I3) involvement of surgical patients.

Indicator Level	Name of the Indicator Level	Indicator Level Description
1	Surgical patient education	The education of orthopedic surgical patients takes place in the preoperative, surgical, and recovery phases, including with the support of electronic health apps.
2	eConsent	In clinical care, patient consent is obtained with the support of digital tools. The digital consent system is in a robust data infrastructure. Patient feedback is collected using surveys, which is processed with the support of the data infrastructure.
3	Shared decision making	Patients are incentivized to get involved in shared decision making. Based on patient feedback, measures to improve medical services are developed and discussed with them.
4	Minimizing damage to normal tissue	Patients are referred for procedures to minimize injury to normal tissue. Microdecompression techniques are used in procedures performed with small incisions. Minimally invasive decompression of the cervical spine uses the cervical microendoscopic foraminotomy and cervical microendoscopic diskectomy procedures.
5	Non-technical skills	The organizational system identifies physicians who, in addition to professional skills, have well-developed, non-practical skills, and patients prefer them to perform medical interventions. The degree of patient satisfaction is high.

**Table 4:** The indicators for (I4) minor surgical interventions.

Indicator Level	Name of the Indicator Level	Indicator Level Description
1	Office-based minor surgery	Minor surgical procedures are performed in the outpatient setting and are accredited. Small skin incisions, minimized muscle dissection, improved visibility with endoscopy and fluoroscopy, application of special instruments, and robotic assistance are practiced.
2	Resident-run minor surgery	Surgical residents independently perform outpatient procedures without jeopardizing patient safety or satisfaction. Biologic osteosynthesis is used in the management of orthopedic trauma.
3	Diagnostic agreement	In the initial diagnosis of lesions referred for evaluation in primary care, there is a high correlation between the clinical diagnosis and the histologic findings that are obtained with the support of minor surgery.
4	Patient satisfaction in minor surgery	Patients are satisfied with the minor surgery, the explanations of anatomopathologic concordances in primary care, and the hygiene of the doctor’s office.
5	Efficiency of minor surgery	In primary care, the minor surgery program has lower costs compared to specialty surgery. The minor surgery program reduces patients’ waiting lists.

**Table 5:** Indicator matrix for remote care pathways in orthopedic surgery.

Level → Indicator ↓	Level 1	Level 2	Level 3	Level 4	Level 5
Remote consultations in orthopedics (I1)	Local clinics	Telephone consultations	Videoconference consultations	Virtual clinic	Orthopedic telesurgery
Incentives for staff travel options (I2)	Active travel	Changing room and shower facilities	Bicycle storage	Hospital parking	Car sharing
Involvement of surgical patients (I3)	Surgical patient education	eConsent	Shared decision making	Minimizing damage to normal tissue	Non-technical skills
Minor surgical interventions (I4)	Office-based minor surgery	Resident-run minor surgery	Diagnostic agreement	Patient satisfaction	Efficiency of minor surgery

**Table 6:** Self-assessment tool for remote care pathways in orthopedic surgery.

No.	Indicator Description	Level (Li)	Importance (Ii)	Sustainability Indicator (Si = Li · Ii)
1	(I1) Remote consultations in orthopedics	3	3	9
2	(I2) Incentives for staff travel options	2	4	8
3	(I3) Involvement of surgical patients	3	2	6
4	(I4) Minor surgical interventions	4	5	20

Li—Level, Ii—Importance, Si—Sustainability Indicator.

## Data Availability

The data used in this study can be requested from the corresponding author.
